# How can we reduce psychological burden for patients of amyotrophic lateral sclerosis and their family caregivers? – Insights from the participatory multi-method study “potentiALS”

**DOI:** 10.1186/s12883-025-04440-w

**Published:** 2025-10-07

**Authors:** Svenja Heyne, Adelina Kuzmanova, Peter Esser, Katharina Linse, René Günther, Anja Mehnert-Theuerkauf, Moritz Metelmann

**Affiliations:** 1 Department of Medical Psychology and Medical Sociology, University Medical Center Leipzig, Philipp-Rosenthal-Str. 55, Leipzig, 04103 Germany; 2 Klinik Angermühle GmbH, Psychosomatic Clinic, Deggendorf, Germany; 3 University Hospital Carl Gustav Carus at Technische Universität Dresden, Dresden, Germany; 4 German Center for Neurodegenerative Diseases Dresden, Dresden, Germany; 5 Department of Neurology, University Medical Center Leipzig, Leipzig, Germany

**Keywords:** Amyotrophic lateral sclerosis, Psychotherapy, Supportive care, Participatory approach, Family caregivers

## Abstract

**Background:**

Amyotrophic lateral sclerosis (ALS) is a progressive, fatal motor neuron disease that severely impacts patients’ physical and emotional well-being, while also imposing significant burdens on family caregivers. Despite the high psychosocial demands as part of the multidimensional burden, evidence for effective interventions remains limited. This study employed a participatory approach to assess the support needs of ALS patients and caregivers and to evaluate their preferences for psychosocial therapies to derive a therapeutic framework.

**Methods:**

In this observational multi-method study, ALS patients, their family caregivers, and healthcare professionals (HCPs) were actively involved throughout the research process. Quantitative Data were collected through structured questionnaires assessing quality of life (e.g., MQoL, SEIQoL-Q), depression and anxiety (ADI-12, HADS), and caregiver burden (BSFC-s). Feedback was obtained through structured group sessions that combined brief introductions, practical exercises, and subsequent evaluations of four therapy approaches – cognitive behavioral therapy (CBT), psychodynamic therapy (PT), acceptance and commitment therapy (ACT), and meaning-centered therapy (MCT).

**Results:**

14 patients, 17 caregivers and nine HCPs participated in the study. Among patients, ACT was the most frequently selected (37.5%), followed by CBT (31.3%), MCT (18.8%), and PT (12.5%) with similar distribution in caregiver attendance. Across all therapy approaches, both patients and caregivers rated the beneficial aspects highly (mean scores of 4.18, and 4.12, respectively, on a scale from 1 to 5) and identified relatively few limitations (mean scores of 2.18 and 2.09, respectively, on a scale from 1 to 5). HCPs corroborated these findings, noting that while the therapies were effective in offering emotional support and facilitating open dialogue, challenges such as time constraints and adapting interventions for speech limitations remain. Notably, caregivers showed a strong preference for individualized therapy, while patients favored a mix of individual and group formats.

**Conclusions:**

Our study highlights the distinct yet interconnected psychosocial needs of ALS patients and their caregivers. Tailored interventions should blend emotional support, open dialogue, and a structured therapeutic framework, while also emphasizing the need for adaptable delivery models in clinical practice. These findings support the development of scalable, patient-centered psychosocial support approaches as part of the multidimensional care in ALS.

**Trial registration:**

The trial is registered at ClinicalTrials.gov (number: NCT06441448, registration date: May 28, 2024).

**Supplementary Information:**

The online version contains supplementary material available at 10.1186/s12883-025-04440-w.

## Background

Amyotrophic lateral sclerosis (ALS) is a progressive and irreversible motor neuron disease (MND) that causes widespread damage to the nervous system, leading to symptoms such as muscle weakness, cramps, pain, pseudobulbar affect, and speech impairments [1, 2]. ALS is invariably fatal, with most patients dying within 3–5 years of symptom onset due to ventilatory failure [1].

The substantial symptom burden and the fatal course of the disease place patients under considerable distress. The prevalence of depression in ALS patients ranges from 10% to 45% [3–5], often accompanied by significant reductions in quality of life (QoL) [6, 7]. Emotional distress, including lability, depression, and anxiety, has been associated with accelerated disease progression in affected individuals [8, 9].

Alongside the physical and psychological burden on patients, ALS imposes substantial demands on family caregivers. As patients lose autonomy in daily activities, caregivers take on increasing responsibility for essential yet complex care tasks, such as feeding, dressing, and mobility support. Approximately 80% of ALS patients rely primarily on their partners for caregiving, which has led to ALS being described as a “family disease” [10]. Family caregivers often experience significant psychosocial stress [11, 12], which is frequently associated with symptoms of depression and anxiety, indicating a substantial caregiving burden [13–15].

Given the complex challenges associated with the disease, individuals with ALS and their caregivers exhibit multifaceted needs, with psychosocial needs appearing to be particularly prevalent [16, 17]. Accordingly, the provision of supportive care that adequately addresses psychosocial issues is warranted [18, 19]. Beyond general support measures, more specific interventions, such as psychotherapeutic treatments, may offer additional benefit in addressing psychological distress.

However, despite the recognized need for such support, empirical evidence regarding the effectiveness of psychosocial interventions for ALS patients and their caregivers remains limited. While one study demonstrated promising outcomes for Acceptance and Commitment Therapy combined with standard care in maintaining or improving QoL among individuals with MND [20], other studies have highlighted methodological limitations that prevent definitive conclusions about effective interventions [21, 22]. Although psychosocial interventions have shown potential benefits, adaptations sensitive to the unique disease context are necessary to improve their acceptability, effectiveness, and engagement [23].

Given the gaps in evidence and the diverse psychosocial needs of ALS patients and family caregivers, this study uses participatory research to generate novel insights that may inform the future development of tailored psychosocial interventions. Participatory methods are particularly promising in this context [23], as they actively engage patients and caregivers as equal partners, grounding intervention design in real-world experiences. By fostering collaboration and mutual learning among all stakeholders, this approach enhances the relevance, acceptability, and feasibility of interventions, ultimately supporting more effective and sustainable outcomes [24]. Based on these methodological advantages, the present study aims to generate practice-oriented insights that directly reflect the expressed preferences and needs of individuals affected by ALS. These findings will serve as a foundation for the systematic development of a research-based, tailored psychosocial intervention that addresses the unique challenges faced by this population.

Therefore, the present study adopts a participatory approach to (i) explore the psychosocial needs of patients with ALS and their family caregivers, and (ii) assess the preferences of patients, caregivers, and healthcare professionals (HCPs) regarding the format, content, and methods of a future psychosocial intervention.

## Methods

### Design and procedure

This observational, multi-method study was conducted in the Departments of Neurology, Medical Psychology and Medical Sociology at the University Medical Center Leipzig, Germany from January 2024 to December 2024. A participatory approach was employed, involving ALS patients, their caregivers, and HCPs engaged in ALS care in the conceptualization of the entire study process.

We employed various methods to reach out to patients, caregivers and HCPs. HCPs were invited to participate in the study through the neurologist’s professional networks. Patients and their caregivers were contacted by the treating neurologists through specialized ALS outpatient clinics in Leipzig and Dresden (Saxony) during regular medical consultations. Additionally, interest was generated through podcasts and various local ALS-specific interest groups.

The inclusion criteria for all participants were: (i) age ≥ 18 years, (ii) fluency in German, and (iii) no behavioral or cognitive impairments that would affect the ability to make judgments or provide informed consent. For patients, additional inclusion criteria were: (i) a diagnosis of possible, probable, or definite ALS according to the revised El Escorial criteria [25], and (ii) absence of percutaneous endoscopic gastrostomy or invasive ventilation. For family caregivers, an additional criterion was: (i) having current or prior experience as a family caregiver for a patient meeting the aforementioned criteria. For HCPs, an additional criterion was: (i) experience in the clinical management of ALS patients.

If patients, their caregivers and HCPs agreed, the study coordinator called them to inform them in detail about the study; subsequently, they could then decide whether or not to participate. The baseline assessment questionnaires were then provided either as paper-pencil versions sent by post or electronically via email, according to individual preferences.

All participants provided written informed consent before study participation. The study has been performed in accordance with the Declaration of Helsinki and was approved by the Ethics Committee of the Medical Faculty of the University of Leipzig (number: 404-23-ek) and is registered at ClinicalTrials.gov (NCT06441448).

### Group session format and data collection

An introductory session was held prior to the group sessions to outline study procedures, clarify participant roles, and assess initial psychosocial and emotional needs. Participants shared their needs using a whiteboard tool within the BigBlueButton platform, which allowed for real-time visualization and collection of inputs from online and on-site participants.

Subsequently, structured group sessions were conducted with patients, family caregivers, and HCPs. At the beginning of each session, the therapist introduced key concepts from four psychotherapeutic approaches: Cognitive Behavioral Therapy (CBT) [26], Acceptance and Commitment Therapy (ACT) [27], Meaning-Centered Therapy (MCT) [28], and Psychodynamic Therapy (PT) [29]. A brief overview of the four therapy approaches is provided in Supplementary Table S1.

Practical exercises were introduced in the group setting but completed individually by participants. Subsequently, participants engaged in a joint reflection on their experiences and provided structured feedback regarding feasibility, perceived helpfulness, and personal preferences through a brief questionnaire. Data collection was thus embedded within the sessions, with a focus on participants’ evaluations and preferences.

Each participant could attend two to four sessions (90 min each), selecting them based on short videos introducing each therapy approach. Sessions were conducted in a hybrid format, allowing participation either on-site or via secure videoconferencing, depending on individual needs and feasibility.

**Fig. 1:**
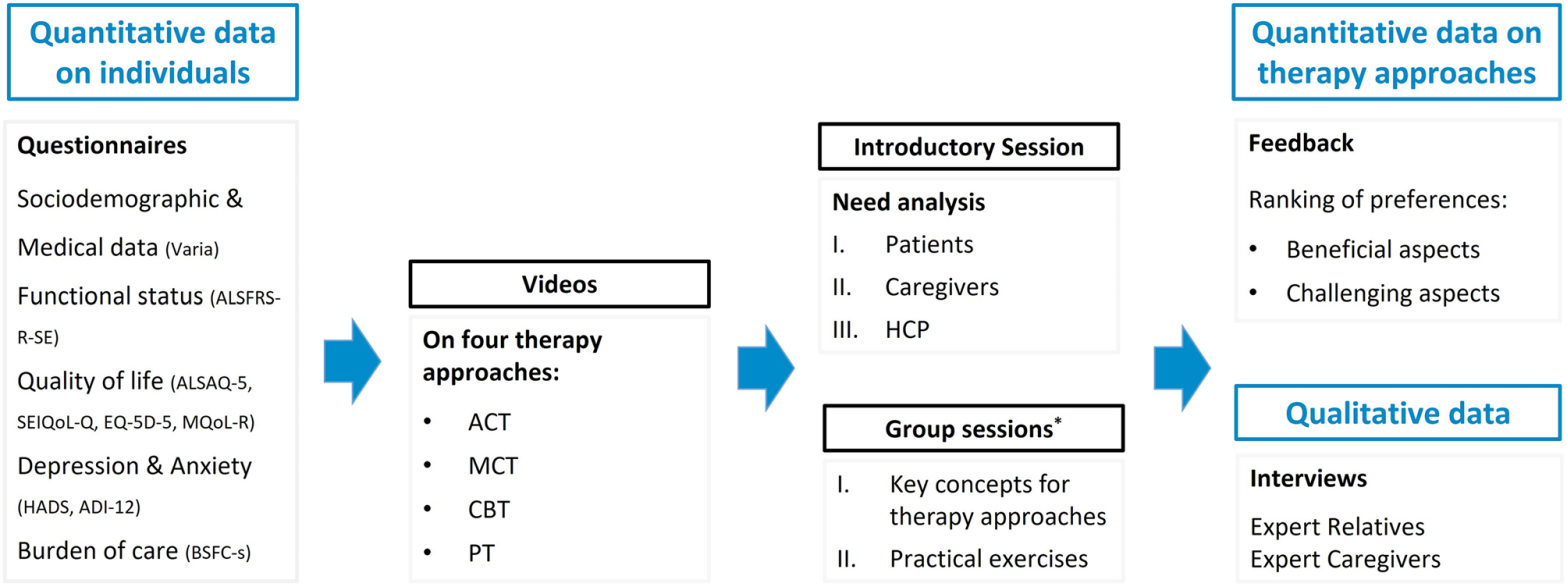
Study Flow with study measures. ALSFRS-R-SE: ALS Functional Rating Scale – Revised – Self-Explanatory [30], ALSAQ-5: Amyotrophic Lateral Sclerosis Assessment Questionnaire - 5 item [31], SEIQoL-Q: Schedule for the Evaluation of Individual Quality of Life – Questionnaire [32], EQ-5D-5: EuroQol 5-Dimension 5-Level [33], MQoL-R: McGill Quality of Life Questionnaire – Revised [34], HADS: Hospital Anxiety and Depression Scale [35], ADI-12: ALS-Depression-Inventory [36], BSFC-s: Burden Scale for Family Caregivers - Short Version [37], ACT: Acceptance and Commitment Therapy, MCT: Meaning-Centered Therapy, CBT: Cognitive Behavioral Therapy, PT: Psychodynamic Therapy, HCP: health-care professionals,^*^group sessions were conducted to gather participant feedback in a structured format; discussions were documented but not subjected to formal qualitative analysis

The complete overview of the study flow is presented in Fig. 1.

### Measures

#### Patient- and caregiver-reported outcome measures

At baseline, quality of life (ALSAQ-5 [31], SEIQoL-Q [32], EQ-5D-5 L [33], MQOL-R [34]), depressive symptoms (HADS [35], ADI-12 [36]), physical functioning (ALSFRS-R-SE [30]), and caregiver burden (BSFC-s [37]) were assessed. For details on the instruments and scoring, see Supplementary Table S2.

#### Functional involvement and disease progression

Data on functional region involvement and the need for interventions were collected to assign each patient to the appropriate King’s stage at baseline with stage 1 (involvement of one region), stage 2 (involvement of two regions), stage 3 (involvement of three regions), and stage 4a (requirement of gastrostomy) or 4b (requirement of non-invasive ventilation) [38]. Disease progression was quantified using ALSFRS-R-SE slopes. The early ALSFRS-R-SE slopes were calculated by subtracting the ALSFRS-R-SE score at the time of the first consultation from 48, and then dividing the result by the time elapsed between the onset of symptoms and the first consultation [39].

#### Feedback from group sessions regarding four therapy approaches

To evaluate perspectives on the four therapeutic approaches (CBT, PT, ACT, MCT) we conducted group sessions with patients, caregivers, and HCPs. Feedback from these groups centered on identifying beneficial and challenging aspects associated with each therapy approach. Participants were asked to evaluate perceived beneficial aspects and challenging aspects of each approach on a self-developed five-point Likert scale ranging from one to five, with higher scores indicating greater approval. The scales were developed based on the needs assessed in the introductory session. We aligned the identified needs with the capabilities of each therapy approach to address them, while also considering any specific challenges. This structure allowed for iterative, reflective feedback, helping to capture evolving perceptions and insights into the therapy approaches over multiple sessions. Additionally, we measured attendance among patients, caregivers and HCP across the four therapeutic approaches as indicator of popularity.

### Statistical analyses

We present descriptive statistics to summarize baseline characteristics and feedback from group sessions. The utility index value from EQ-5D-5 L is calculated by subtracting the respective utility decrements associated with each level of severity in the five dimensions from one (representing full health). The specific decrements for each level and dimension are detailed in the German EQ-5D-5 L value set study [40].

Data analysis was performed using SPSS Version 29 [41] with frequencies, means, and standard deviations calculated for continuous and categorical variables. Group comparisons were made where appropriate, and all data were evaluated for completeness and consistency.

## Results

### Participant flow

**Fig. 2:**
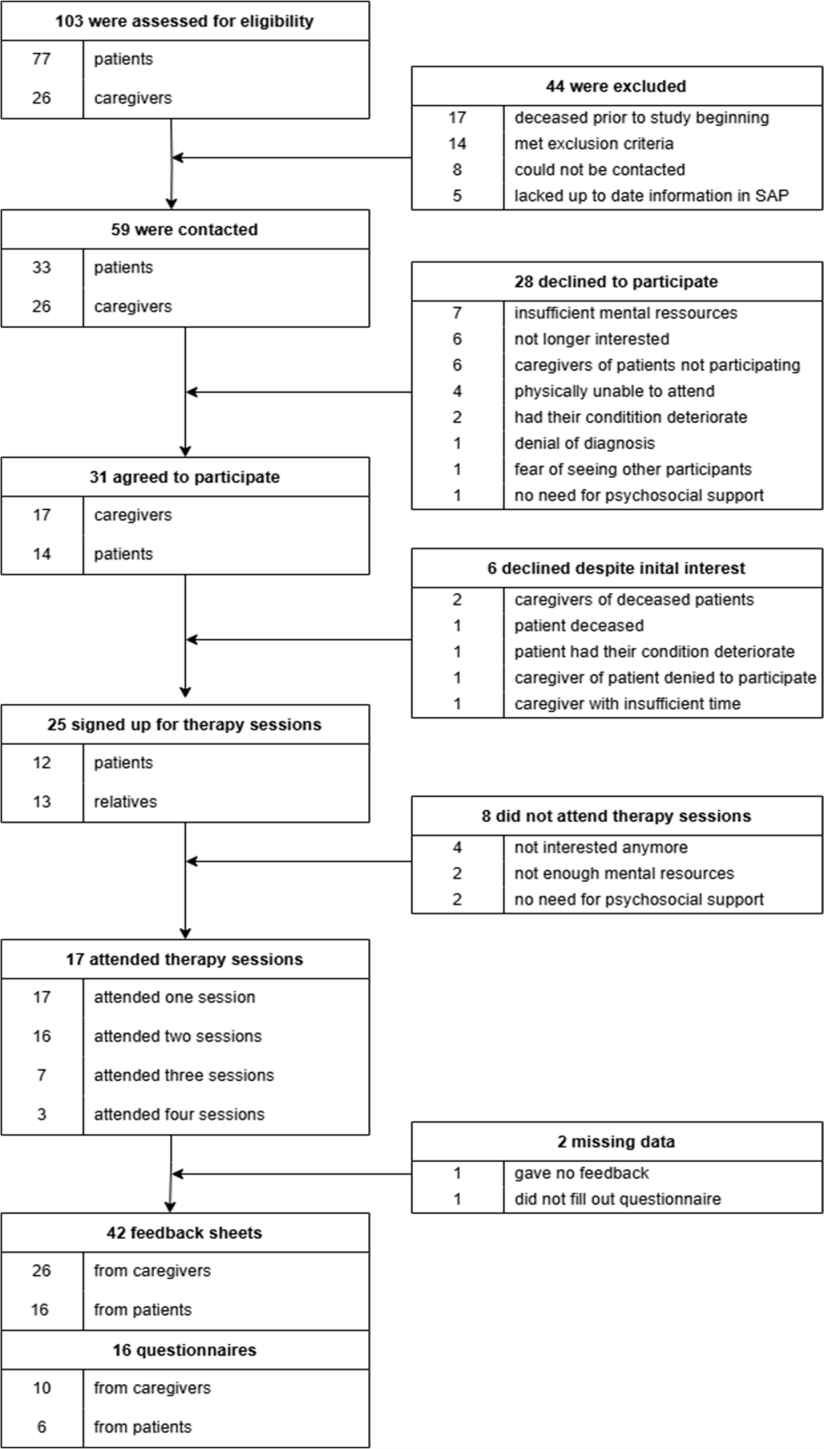
Flowchart patients and caregivers

A total of 103 individuals were assessed for eligibility. Details regarding participant flow, including numbers contacted, reasons for non-participation, and attendance at therapy sessions, are summarized in Fig. 2. For the final analysis, feedback from therapy sessions (*n* = 42) and questionnaires on sociodemographic, psychosocial, and ALS-specific data (*n* = 16) were included.

**Fig. 3:**
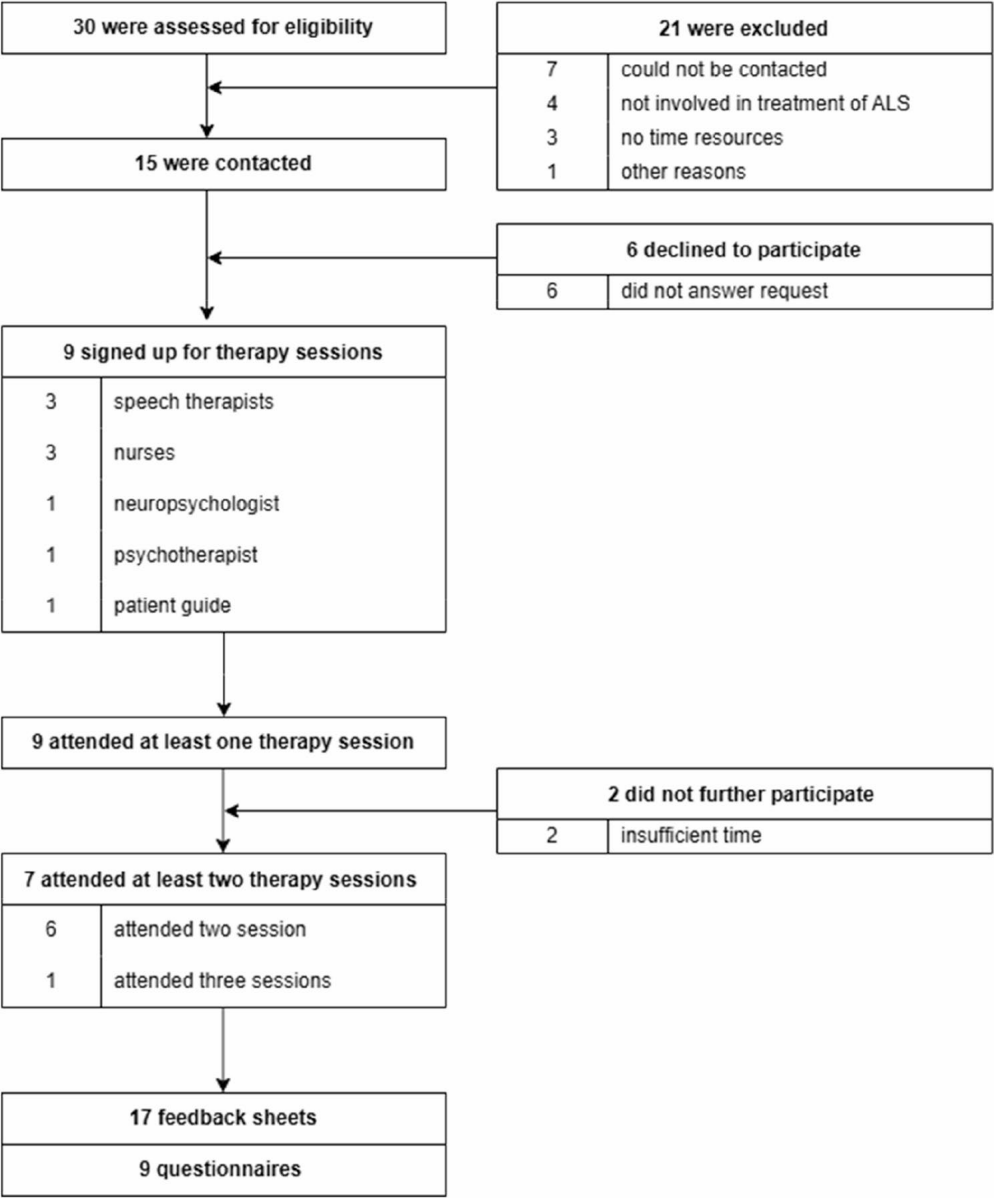
Flowchart health care professionals

A total of 30 HCPs were assessed for eligibility. Participant flow, including numbers contacted, reasons for non-participation, and session attendance, is summarized in Fig. 3. For the final analysis, feedback from therapy sessions (*n* = 17) and questionnaires on sociodemographic data and ALS expertise (*n* = 9) were included.

### Baseline characteristics

The median age of patients was 66.0 years (IQR = 9.0), while the median age of caregivers was 56.0 years (IQR = 29.0). The duration since symptom onset ranged from one month to 15 years. All patients (100.0%) were diagnosed with definite, sporadic ALS. Of these, 4 patients (57.1%) had spinal onset, while the remaining had bulbar onset. Table 1 provides a comprehensive summary of the baseline characteristics of both patients and caregivers.

**Table 1:** Baseline sociodemographic characteristics for patients and caregivers

	Patients	Caregivers
	*n* (%)	*n* (%)
	7 (100.0)	10 (100.0)
Sociodemographic data		
Sex		
Male	5 (71.4)	-
Female	2 (28.6)	10 (100.0)
Marital status		
Single	-	2 (20.0)
Married	6 (85.7)	6 (60.0)
Divorced	1 (14.3)	2 (20.0)
Living with a partner		
Yes	6 (85.7)	9 (90.0)
Having children		
Yes	7 (100.0)	7 (70.0)
Household income in €/month^a^		
1.500–2.499	2 (28.6)	-
2.500–3.500	3 (42.9)	4 (40.0)
over 3.500	2 (28.6)	5 (50.0)

*n* sub-sample size, ^a^NA=1

In terms of caregiver-patient relationships, seven caregivers (70.0%) were spouses, two (20.0%) were sons or daughters, and one (10.0%) was another family member. Table 2 provides an overview over the baseline medical characteristics for patients.

**Table 2:** Baseline medical characteristics for patients

	Patients
	*n* (%)
Medical data	
Care level	
Yes	3 (42.9)
No	4 (57.1)
Riluzole	
Yes	5 (71.4)
No	2 (28.6)
NIV	
Yes	2 (28.6)
No	5 (71.4)
ALS progression rate^1, a^	
Slow (< 0.47 points/month)	2 (33.3)
Intermediate (0.47–1.11 points/month)	2 (33.3)
Fast (> 1.11 points/month)	2 (33.3)
Functional region involvement ^2^	
Stage 1	1 (16.7)
Stage 2	3 (33.3)
Stage 3	-
Stage 4a	-
Stage 4b	3 (50.0)

*n* sub-sample size, *NIV* Non-invasive ventilation

^1^calculated via ALSFRS-R-SE slopes and categorized according to [39], ^2^indicated by King’s stage [38], ^a^missing value=1

The median age of HCPs was 38.0 years (IQR = 24.0). The majority were female (88.9%). HCPs can be categorized into four professional roles: speech therapists, research associates, psychologists and psychological psychotherapists and nursing professions. With regard to familiarity with ALS, five (55.6%) HCPs were fairly familiar with the condition, two (22.2%) were very familiar, and two (22.2%) were minimally familiar. The median number of years working with ALS patients was 9.0 years (IQR = 10.0). Five (55.6%) HCPs cared for fewer than five ALS patients annually, two (22.2%) cared for five to fewer than ten patients, and two (22.2%) cared for ten to twenty patients each year.

### Baseline psychological and quality of life scores for patients and caregivers

Most patients (83.3%) showed mild depressive symptoms. Among caregivers, 54.5% had no significant distress, while the rest reported mild to clinically significant anxiety and/or depression (18.2–27.3%). Table 3 provides an overview of psychological and functional status, quality of life, and caregiver-related factors, such as caregiver burden at baseline.

**Table 3:** Baseline psychological, functional status and quality of life scores for patients and caregivers

Measures	Patients			Caregivers		
	Mean	SD	Range	Mean	SD	Range
Functional status						
ALSFRS-R-SE [30]	33.83	5.98	25–42	N/A	N/A	N/A
Depression and anxiety						
ADI-12 [36]	21.83	0.98	20–23	N/A	N/A	N/A
HADS-A [35]	6.00	4.29	0–12	7.27	3.63	1–12
HADS-D [35]	6.17	3.25	4–12	7.45	2.91	4–12
Quality of life						
ALSAQ-5[31]	40.00	24.91	20–72	N/A	N/A	N/A
EQ-5D - Index Score [33]	0.42	0.56	−0.66−0.91	0.84	0.08	0.74–0.92
EQ-5D - VAS Score [33]	50.83	33.22	10–90	82.00	9.11	62–95
MQoL - Physical WB [34]	6.47	1.82	5–9	8.03	1.92	4–10
MQoL - Emotional WB [34]	6.88	1.19	6–8	6.15	1.80	4.38–9
MQoL - Support [34]	8.93	1.73	6–10	7.36	2.69	2.33–10
MQoL - Global QoL [34]	5.67	2.33	3–9	6.80	2.30	3–9
SEIQoL-Q - Global QoL [32]	55.60	9.40	48.61–73.44	52.81	19.06	15.63–83.09
Caregiver Burden						
BSFC - Global Score[37]	N/A	N/A	N/A	19.88	8.82	0–30

*SD* Standard deviation, *N/A* Not applicable, *WB* Well-being, *QoL* Quality of life, *VAS * Visual Analogue Scale, *ALSFRS-R-SE* ALS Functional Rating Scale – Revised – Self-Explanatory, *ADI-12* ALS-Depression-Inventory, *HADS *Hospital Anxiety and Depression Scale, *ALSAQ-5* Amyotrophic Lateral Sclerosis Assessment Questionnaire - 5 item, *EQ-5D-5* EuroQol 5-Dimension 5-Level, *MQoL-R *McGill Quality of Life Questionnaire – Revised,* SEIQoL-Q* Schedule for the Evaluation of Individual Quality of Life – Questionnaire, *BSFC-s* Burden Scale for Family Caregivers - Short Version

### Assessment of needs for patients and caregivers

For the needs assessment, 36 individuals participated, comprising 11 patients, 11 caregivers and 14 HCP. Table 4 provides a detailed summary of the initial assessment of psychosocial needs, highlighting both the common and unique requirements of patients and caregivers, as perceived by themselves and evaluated by HCP.

**Table 4:** Summary of psychosocial needs assessment

	Self-evaluation from patients and caregivers			External evaluation from HCP		
Category	Shared Needs (Patients & Caregivers)	Patient-Specific Needs	Caregiver-Specific Needs	Shared Needs (Patients & Caregivers)	Patient-Specific Needs	Caregiver-Specific Needs
Social care	• Administrative assistance (e.g., appeals for aid rejections) • Assistance in maintaining household and daily activities	• Retaining meaningful connections and support from social networks	• Support for maintaining a sense of normalcy (e.g. hobbies, work)	• Exchange and a sense of not being alone		
Information & Counseling	• Counseling on clinical trials and research findings • Personalized, non-generic information delivery • Comprehensive disease information	• Early provision of disease-specific information • Development and use of an ALS-related app		• Modifying content of information based on individual needs		• Tailored information to manage caregiving tasks
Psychological Support	• Psychological support, especially at the beginning • Psychological services for families with children • Focus on fears, grief, and coping mechanisms • Joint psychotherapy sessions for patients and caregivers • Needs-based support in various life situations	• Support in managing fears • Support in managing grief • Support in adjusting to a new self-image • Emotional strategies for maintaining positivity despite prognosis	• Support in fostering resilience • Coping with personal fears and uncertainties • Emotional support to prevent feelings of abandonment	• Psychological support, especially at the beginning and during the diagnosis process • Need for short-term interventions, e.g., crisis intervention	• Support in managing feelings of anxiety	• Support in managing feelings of abandonment
Quality of Life	• Creating coping strategies for lifestyle adjustments	• Continuing professional and social activities as long as possible • Pursuing modified hobbies or travel opportunities	• Balancing caregiving responsibilities with personal life and work		• Coping with helplessness as the body “deteriorates” despite intact cognitive abilities	• Pursuing a sense of balance in caregiving roles
Participation		• Discussion of medically assisted suicide			• Support in decision-making regarding therapy options • Assistance in addressing end-of-life topics	

*ALS* Amyotrophic lateral sclerosis

### Feedback regarding the four therapy approaches

Among patients, ACT (6, 37.5%) was the most frequently attended therapy, followed by CBT (5, 31.3%), MCT (3, 18.8%), and PT (2, 12.5%). Among caregivers, attendance was highest for both ACT and CBT (8, 26.9%), followed by MCT (7, 25.9%), and PT (4, 14.8%).

**Fig. 4:**
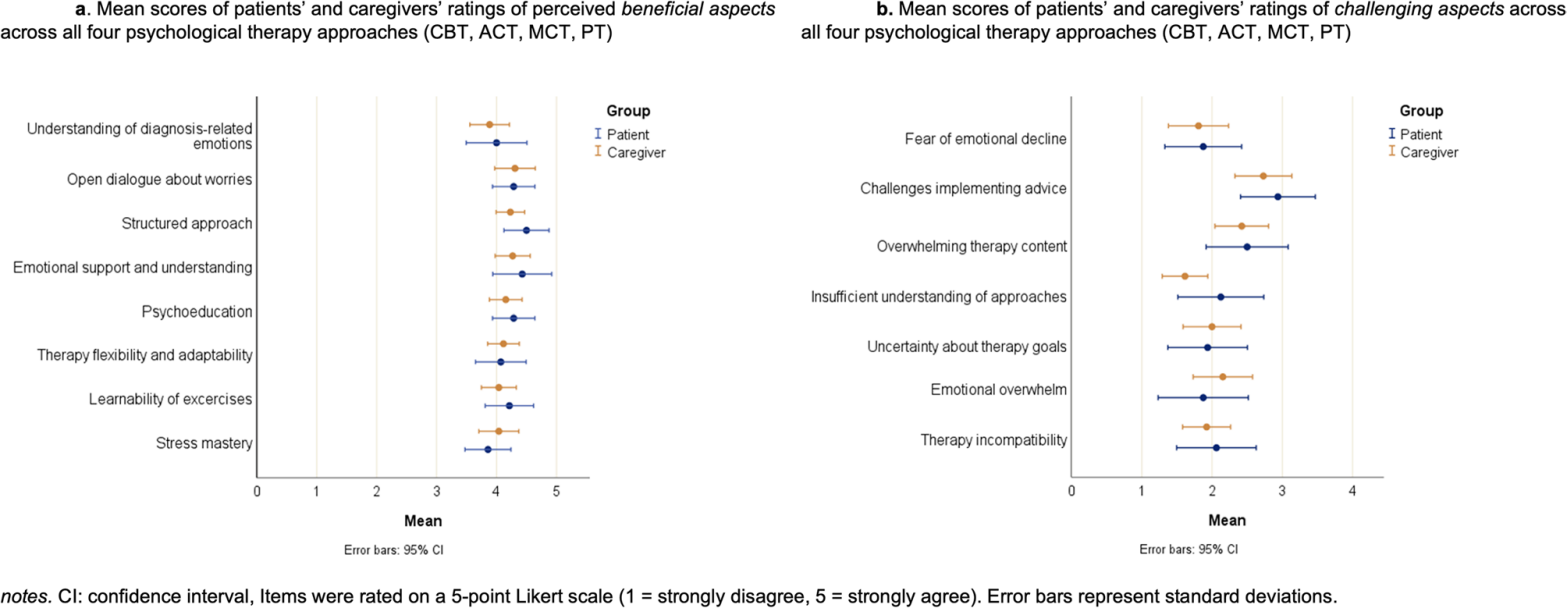
Perceived beneficial (**a**) and challenging aspects (**b**) of psychological therapy approaches as rated by patients and caregivers

Mean scores for beneficial aspects across all therapy approaches were high for both groups: patients, 4.18 (± 0.39, range: 3.50–4.75) and caregivers, 4.12 (± 0.49, range: 3.25–5.00). Mean scores for challenging aspects across all therapy approaches were low: patients, 2.18 (± 0.78, range: 1.14–3.57) and caregivers, 2.09 (± 0.69, range: 1.00–3.57). Detailed distributions and item scores are presented in Fig. 4a and b.

**Fig. 5:**
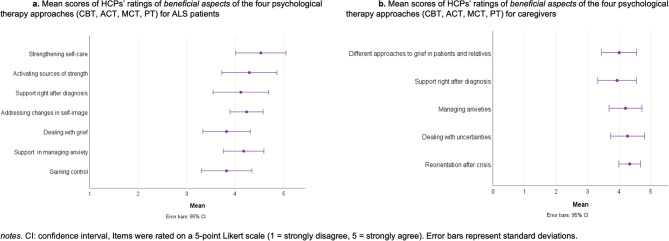
Perceived beneficial aspects of psychological therapy approaches: healthcare professionals’ ratings for patients with ALS (**a**) and their caregivers (**b**)

Among HCPs, attendance was highest for both ACT and CBT (5, 29.4%), followed by MCT (4, 23.5%), and PT (3, 17.6%). Mean scores for beneficial aspects across all therapy approaches were high: 4.17 (± 0.70, range: 2–5) for patients and 4.14 (± 0.64, range: 2–5) for caregivers, as rated by HCPs. Detailed distributions and item scores are presented in Fig. 5a and b.

Ten (58.8%) HCP reported specific challenges such as time constraints due to the rapid progression of the disease, limited adaptability for speech issues, and difficulty integrating therapies into routine practice.

Overall, an individual setting was favored by most participants (30, 71.4%), especially caregivers (23, 88.5%), while group settings were favored by a minority (3, 11.5%). Among patients, preferences were more evenly split between individual (7, 43.7%) and group settings (9, 56.3%).

## Discussion

This study highlights the importance of addressing the psychosocial needs of ALS patients and their family caregivers. By actively involving these people affected alongside healthcare professionals, we were able to identify key priorities and preferences for a future psychosocial intervention tailored to their specific needs and experiences.

### Main findings and integration into existing literature

Patients demonstrated mild levels of depression on the ADI-12 alongside moderate functional impairment (ALSFRS-R-SE score = 33.83) comparable to a Germany-wide, multicenter cross-sectional study [42]. In contrast, caregivers, despite generally reporting a moderate quality of life, exhibited elevated anxiety and depression levels, echoing findings from a Swedish cohort [43]. Moreover, 70% experienced significant caregiver burden, consistent with previous research highlighting significant caregiver strain [44–47].

We found that ALS patients and caregivers have distinct yet interconnected psychosocial needs, including early psychosocial support at diagnosis, personalized disease information, and assistance with administrative tasks. Evidence from studies on information-seeking behaviors among ALS patients underscores the critical role of disease-specific information in effective symptom management [48]. Furthermore, recent research highlights caregivers’ substantial practical and administrative support needs, including access to information and referral pathways [16, 49], with comparable needs also identified among patients [50]. Patients further emphasized the significance of social connections, aligning with a Chinese study that found social support directly impacts well-being [51]. Caregivers, meanwhile, stressed the need for more personal time to balance caregiving with other responsibilities, reflecting similar concerns from a Dutch study highlighting the necessity of strategies to support caregiver well-being [52].

Regarding the beneficial aspects across all four therapy approaches, both patients and caregivers most valued emotional support and understanding, fostering open dialogue, and a structured therapy approach. HCPs largely aligned with these views but also emphasized strengthening self-care, addressing self-image changes, and supporting caregivers in managing anxieties and crises. Key challenges included difficulties implementing advice, overwhelming therapy content, and emotional strain on caregivers. HCPs highlighted significant barriers to integrating psychosocial interventions, particularly due to ALS progression and speech limitations. However, the barriers to implementation as raised by HCP mirror prior concerns raised in the literature [19, 53, 54].

Regarding setting preferences, the strong inclination of caregivers (88.5%) toward individual support versus the mixed preference among patients (43.7%) is notable. The divergence in preferences reflects the distinct roles and challenges faced by caregivers and patients. Caregivers often juggle dual responsibilities (e.g., caregiving and personal life), leading them to prioritize individual therapy for tailored coping strategies [12]. In contrast, ALS patients may benefit from both peer support in group settings as this can provide a non-judgmental and supportive environment, in which they can exchange experiences and emotional support [55] and individualized support allowing for a more in-detail exploration of thoughts, feelings and circumstances.

### Implications

These findings highlight the need for psychosocial interventions that are structurally organized yet flexible in their implementation, evolving in parallel with the progressive nature of ALS and the corresponding changes in patient and caregiver needs. From our perspective, a structured approach is essential, as both patients and caregivers in our study emphasized the importance of clearly defined therapeutic goals and a coherent intervention framework. At the same time, interventions must allow for flexible, context-specific adaptations, e.g. allowing participants to choose discussion topics or to modify and substitute exercises as needed. Other studies have demonstrated that overly rigid approaches are often unsuitable and may be associated with higher dropout rates [16, 19].

Our study demonstrated the feasibility of actively involving patients and caregivers in the research process. The integration of participatory approaches within the design and refinement of interventions, alongside the promotion of autonomy, has been shown to enhance their feasibility, accessibility, and overall effectiveness [19]. One factor that could facilitate these approaches is the use of e-health tools, such as videoconferencing, which was employed in our study. Further studies should incorporate these elements to create a setting that minimizes emotional exposure for individuals with pseudobulbar affect [56] and other common barriers, like mobility issues, limiting on-site attendance. Our approach aligns with a growing body of literature demonstrating the effectiveness and acceptability of online psychological interventions across various patient populations [57–59].

We did not directly compare the four therapeutic approaches with regard to their specific components or differential effectiveness due to limited sample sizes within each group. However, the beneficial elements identified across approaches, as well as the contextual needs expressed by participants, should guide the development of future psychosocial care frameworks in ALS. Further research should evaluate the efficacy and effectiveness of such frameworks and explore to what extent tailoring psychosocial interventions to the individual preferences of patients and caregivers enhances psychological well-being, uptake, and engagement [19].

Future research should further explore whether psychosocial support in ALS is more effective when delivered through a specific therapeutic modality, or whether non-specific support may be equally beneficial. This question arises from our findings that key beneficial elements, such as open dialogue and emotional support, appear to be valuable across the four therapeutic approaches. Moreover, participants often found it challenging to distinguish between the unique characteristics of the individual therapy modalities; instead, the therapeutic relationship and the provision of emotional care played a more central role in their overall experience. These elements are not specific to any one modality but rather reflect so-called common factors in psychotherapy [60]. Our findings therefore support the notion that such cross-cutting factors may play a central role in meeting the psychosocial needs of ALS patients and their caregivers, independent of the specific therapeutic model.

Our study focused on individual psychotherapeutic models. However, couple- and family-oriented interventions may also be valuable in ALS, given their demonstrated benefits in other chronic conditions (e.g., stroke, cancer, arthritis) [61]. Future research should therefore examine the feasibility and effectiveness of systemic approaches within ALS care.

### Strengths and limitations

By utilizing a participatory, multi-method approach that integrates diverse perspectives, we engaged patients, caregivers, and HCPs to explore the challenges and benefits of various therapy approaches . Group sessions allowed for an in-depth exploration of participants’ attitudes, expectations, and concerns.

Another strength of our study was the balanced distribution of patients across early to mid-stage (Stages 1–2) and late-stage (4b) disease, as well as varying progression rates, ensuring a cohort with diverse disease severity and progression.

However, the study has several limitations. Patients with severe cognitive impairments or those requiring invasive ventilation were excluded to ensure meaningful group participation, potentially omitting perspectives from advanced disease stages with severe impairments. Additionally, recruitment proved difficult despite strong efforts through ALS centers, self-help organizations, and podcasts. Patients, in particular, were harder to enroll, leading to a small sample size that may limit the generalizability of the results. This also led to the decision to evaluate the beneficial and detrimental aspects across all therapy approaches rather than individually in order to draw meaningful conclusions.

## Conclusion

Our study reinforces existing literature on the psychosocial challenges faced by ALS patients and their caregivers, providing nuanced insights into their distinct needs. The results advocate for a psychosocial support as part of multidimensional ALS care. Open dialogue and emotional support were key elements across all four therapeutic approaches and should be central to a future psychosocial intervention that is structured yet flexible. Building on these findings, future research will focus on developing a tailored intervention that integrates individual preferences and needs of patients and caregivers. Rather than adopting an “one size fits all” - model, this intervention should allow for personalized combinations of the therapy approaches evaluated. Further qualitative investigations are planned to refine the intervention components, and subsequent quantitative studies including randomized controlled trials will assess its feasibility and efficacy.

## Supplementary Information

Supplementary Material 1.

Supplementary Material 2.

## Data Availability

The datasets used and analysed during the current study are available from the corresponding author on reasonable request.

## Data availability

The datasets used and analysed during the current study are available from the corresponding author on reasonable request.
