# Supplementary material for: How can we reduce psychological burden for patients of amyotrophic lateral sclerosis and their family caregivers? – Insights from the participatory multi-method study “potentiALS”
Source: BMC Neurol. 2025 Oct 7;25:414. doi: 10.1186/s12883-025-04440-w (PMC12502389; doi:10.1186/s12883-025-04440-w)
Supplement: Supplementary file 1 — Supplementary Material 1. [file 12883_2025_4440_MOESM1_ESM.docx]

| **Table S1.** Overview of the four therapeutic approaches introduced during group sessions | | |
| --- | --- | --- |
| **Therapy Approach** | **Core Principles** | **Therapeutic Focus in ALS Context** |
| **CBT,** Cognitive Behavioral Therapy **[26]** | - Focuses on identifying and changing unhelpful thoughts and behaviors - Uses structured techniques to build coping skills | - Helping patients and caregivers manage anxiety, depressive thoughts, and cognitive distortions related to illness and caregiving |
| **ACT,** Acceptance and Commitment Therapy **[27]** | - Encourages acceptance of difficult experiences and commitment to personal values - Emphasizes psychological flexibility | - Supporting emotional regulation, acceptance of the illness, and reorientation toward meaningful life aspects despite illness |
| **MCT,** Meaning-Centered Therapy **[28]** | - Draws on existential and spiritual dimensions - Helps individuals connect to sources of meaning, identity, and legacy | - Offering patients and caregivers a space to explore meaning, values, and personal legacy in the face of terminal illness |
| **PT, Psychodynamic Therapy [29]** | - Explores unconscious conflicts, past relational patterns, and emotional experiences - Emphasizes the therapeutic relationship | - Fostering emotional awareness, expression, and interpersonal perspective-taking - Working with imagination/visualization |

*notes.* ALS: Amyotrophic Lateral Sclerosis
