# Supplementary material for: How can we reduce psychological burden for patients of amyotrophic lateral sclerosis and their family caregivers? – Insights from the participatory multi-method study “potentiALS”
Source: BMC Neurol. 2025 Oct 7;25:414. doi: 10.1186/s12883-025-04440-w (PMC12502389; doi:10.1186/s12883-025-04440-w)
Supplement: Supplementary file 2 — Supplementary Material 2. [file 12883_2025_4440_MOESM2_ESM.docx]

| **Table S2.** Overview of patient- and caregiver-reported outcome measures | | | |
| --- | --- | --- | --- |
| **Instrument** | **Purpose / Target Group** | **Domains / Dimensions** | **Scoring / Interpretation** |
| **ALSAQ-5** (Amyotrophic Lateral Sclerosis Assessment Questionnaire – 5 item) [31] | Disease-specific QoL for ALS patients | Physical mobility, daily living, eating/drinking, communication, emotional functioning | 5 items, 5-point Likert scale; score 0–100; higher scores = greater impairment |
| **EQ-5D-5L** (EuroQol 5-Dimension 5-Level) [33] | General health status | Mobility, self-care, usual activities, pain/discomfort, anxiety/depression + VAS | 6 items, 5 level scale (no -exteme problems); 5-digit health state code; utility index (-0.661 to 1.000); VAS 0–100 |
| **MQOL-R** (McGill Quality of Life Questionnaire – Revised) [34] | QoL in serious illness/palliative care | Physical well-being, emotional well-being, support, global QoL | 15 items, 10-point scale; total score 0–140; higher = better QoL |
| **SEIQoL-Q** (Schedule for the Evaluation of Individual Quality of Life - Questionnaire) [32] | Individualized QoL assessment | 10 domains (family, partnership, friends, social life, finances, work, physical health, emotional well-being, autonomy, home, hobbies, and spirituality) | Importance and satisfaction ratings (0–100); individual QoL index (0–100); higher = better QoL |
| **ADI-12** (ALS-Depression-Inventory) [36] | Depression screening in ALS | Cognitive and emotional symptoms | 12 items, 4-point Likert; total score 0–48; 22-28 = mild, >28 = clinically relevant depression |
| **HADS** (Hospital Anxiety and Depression Scale) [35] | Screening for anxiety and depression | Anxiety (7 items), depression (7 items) | 14 items, 0–21 per subscale; 0–7 = normal, 8–10 = borderline, 11–21 = abnormal |
| **ALSFRS-R-SE** (ALS Functional Rating Scale – Revised – Self-Explanatory) [30] | Functional status in ALS | Bulbar, fine motor, gross motor, respiratory | 12 items, 0–4 scale; total 0–48; higher = better function |
| **BSFC-s** (Burden Scale for Family Caregivers – Short Version) [37] | Caregiver burden | Physical, emotional, social, financial strain | 10 items,4-point Likert; total 0–30; 0–4 = no/little, 5–14 = moderate, 15–30 = severe burden |

*notes.* ALS: Amyotrophic lateral sclerosis, QoL: Quality of life, VAS: Visual analogue scale
